# Fine‐scale genetic structure and flowering output of the seagrass *Enhalus acoroides* undergoing disturbance

**DOI:** 10.1002/ece3.5106

**Published:** 2019-04-21

**Authors:** Shuo Yu, Yunchao Wu, Ester A. Serrao, Jingping Zhang, Zhijian Jiang, Chi Huang, Lijun Cui, Anitra Thorhaug, Xiaoping Huang

**Affiliations:** ^1^ Fourth Institute of Oceanography Ministry of Natural Resources Beihai China; ^2^ Key Laboratory of Tropical Marine Bio‐resources and Ecology, South China Sea Institute of Oceanology Chinese Academy of Sciences Guangzhou China; ^3^ University of Chinese Academy of Sciences Beijing China; ^4^ CCMAR University of Algarve Faro Portugal; ^5^ Ocean University of China Qingdao China; ^6^ School of Forestry an Environmental Studies Yale University New Haven Connecticut

**Keywords:** clonal diversity, disturbance, *Enhalus acoroides*, seagrass, sexual output

## Abstract

Seagrass are under great stress in the tropical coast of Asia, where *Enhalus acoroides* is frequently the dominant species with a large food web. Here, we investigate the question of the fine‐scale genetic structure of this ecologically important foundation species, subject to severe anthropogenic disturbance in China. The genetic structure will illuminate potential mechanisms for population dynamics and sustainability, which are critical for preservation of biodiversity and for decision‐making in management and restoration. We evaluated the fine‐scale spatial genetic structure (SGS) and flowering output of *E. acoroides*, and indirectly estimated the relative importance of sexual versus asexual reproduction for population persistence using spatial autocorrelation analysis. Results reveal high clonal diversity for this species, as predicted from its high sexual reproduction output. The stronger *Sp* statistic at the ramet‐level compared with genet‐level indicates that clonality increases the SGS pattern for *E. acoroides*. Significant SGS at the genet‐level may be explained by the aggregated dispersal of seed/pollen cohorts. The estimated gene dispersal variance suggests that dispersal mediated by sexual reproduction is more important than clonal growth in this study area. The ongoing anthropogenic disturbance will negatively affect the mating pattern and the SGS patterns in the future due to massive death of shoots, and less frequency of sexual reproduction.

## 1 INTRODUCTION

Seagrasses form one of the most important ecosystems along coastal regions, providing valuable ecosystem services and functions (Orth et al., 2006). However, such ecosystems are under threat due to anthropogenic disturbances (e.g., boating, aquaculture, pollution and coastal reclamations) and climate change, which are causing seagrass habitat loss and fragmentation and even local or regional extinctions (Waycott et al., 2009; Williams et al., 2016). Therefore, seagrass conservation and restoration have become growing concerns globally in ecology (van Katwijk et al., 2016; Williams, Ambo‐Rappe, Sur, Abbotte, & Limbong, 2017). Seagrass species exhibit both clonal and sexual reproduction (Den Hartog, 1970). To better understand the relative importance of clonal versus sexual reproduction within populations is critical for species conservation and ecological restoration and effective management, because the recovery ability of seagrasses after disturbances mainly depend on their reproductive and recruitment strategy (Kendrick et al., 2017; Macreadie, York, & Sherman, 2014).

Previous studies on seagrass recovery after small‐scale disturbances showed that clonal growth is the key recovery mode for the small, fast‐growing species, while seedling recruitments may contribute to recolonization for the large, slow‐growing species (Olesen, Marbà, Duarte, Savela, & Fortes, 2004; Smith et al., 2016). However, in the long term, the relative contribution of clonal and sexual reproduction for population maintenance is more complex, and even variable in different locations for the same species, because it is influenced by gene flow, population history, and local environmental factors (Kendrick et al., 2012).

Pollen and seed dispersal are very difficult to be detected directly in the marine environment. Nevertheless, several studies have proved that historical gene dispersal mediated by propagules (e.g., pollen, seeds, seedlings, and clonal fragments) can be estimated indirectly from the fine‐scale spatial genetic structure of populations at drift–dispersal equilibrium using spatial autocorrelation analysis (Alberto et al., 2005; Hardy et al., 2006; Smouse, Peakall, & Gonzales, 2008). Spatial genetic structure (SGS), the nonrandom spatial distribution of alleles, occurs frequently within sessile plant populations as a consequence of limited dispersal of propagules, mating system, and other ecological factors (Monthe, Hardy, Doucet, Loo, & Duminil, 2017; Vekemans & Hardy, 2004; Wang, Compton, Shi, & Chen, 2012). Gene flow mediated by pollen and seed is the key factor in determining the SGS pattern within and among populations (Aguilar, Quesada, Ashworth, Herrerias‐Diego, & Lobo, 2008; Wang, Compton, & Chen, 2011). For clonal plants (e.g., seagrasses), clonal growth also profoundly influences the ramet‐level and genet‐level SGS patterns (Arnaud‐Haond, Duarte, Alberto, & Serrao, 2007; Procaccini, Olsen, & Reusch, 2007). Clonal growth is a component of gene dispersal through rhizome elongation, and the clumped clone mates lead to aggregated distribution for certain alleles, resulting in the ramet‐level SGS (Alberto et al., 2005; Becheler, Benkara, Moalic, Hily, & Arnaud‐Haond, 2014). In addition, clonal reproduction may change the mating patterns, such as by increasing geitonogamy (Reusch, 2001), or may constrain sexual reproduction because of resource allocation trade‐offs (Vallejo‐Marín, Dorken, & Barrett, 2010). Those effects of clonality on sexual reproduction can indirectly influence the genet‐level SGS. Consequences of disturbance on SGS for clonal plants are complicated, and poorly understood, because they depend on the intensity and scale of the disturbance, the type of disturbance, and the feedbacks or resilience within populations (Banks et al., 2013; Hughes, Byrnes, Kimbro, & Stachowicz, 2007; Reusch, 2006).

*Enhalus acoroides*, a large dioecious seagrass, exhibits very slow rhizome elongation rate (Marbà & Duarte, 1998; Thorhaug & Cruz, 1986), but relatively high sexual production (Duarte et al., 1997). It flowers throughout the year and pollinates on the water surface (Ackerman, 2006). The plant forms a large fruit pod, within which an average of 11.8 ± 4.04 seeds are contained. Either the fruit dehisces in place with the seeds sinking to the bottom or if the plant releases the fruit, which may on occasion float up to kilometers following the currents (Kendrick et al., 2012; Lacap, Vermaat, Rollon, & Nacorda, 2002). In China, *E. acoroides* occurs along the east coastline of Hainan Island as a dominant species in seagrass communities, forming continuous monospecific beds or mixed meadows with other seagrass species, such as *Halophila ovalis*, *Thalassia hemprichii*, and *Cymodocea rotundata*. Unfortunately, the distribution area and aboveground cover of *E. acoroides* have declined and been fragmented due to both a high level of pollution and physical disturbances (Chen et al., 2015; Herbeck, Sollich, Unger, Holmer, & Jennerjahn, 2014; Yu et al., 2018). However, genetic impacts are not readily visible in the phenotypes; therefore, the fine‐scale spatial genetic pattern of *E. acoroides* populations remains unclear. In this study, we addressed two main objectives: (a) to evaluate the clonal diversity, fine‐scale spatial genetic structure (SGS), and flowering output of *E. acoroides* and (b) to estimate the relative contribution of clonal growth and sexual reproduction to the gene dispersal. These data will be very useful for conservation and restoration actions for *E. acoroides*.

## 2 MATERIALS AND METHODS

### 2.1 Study sites and sampling design

**Figure 1:**
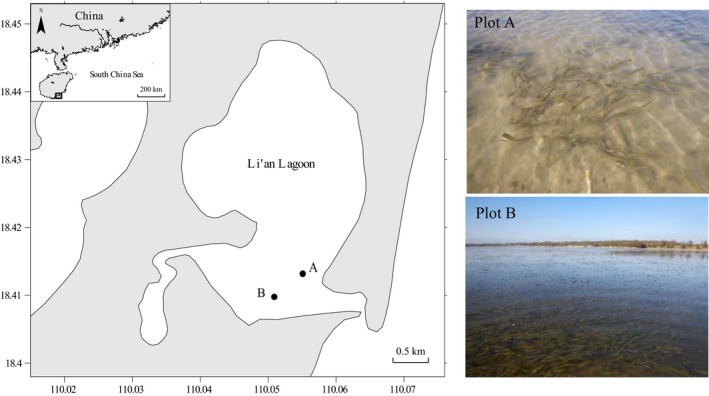
Sampling sites of *Enhalus acoroides* in Li'an Lagoon

To assess the fine‐scale genetic structure of *E. acoroides*, we designated two 20 × 20 m plots in Li'an lagoon (LA) in October 2016 (Figure 1). The seagrass meadow is about 10.1 km^2^ dominated by *E. acoroides* in LA lagoon, other species including *T. hemprichii*, *C. rotundata*, and *H. ovalis*. However, the seagrass distribution area has declined and become fragmented since 1995 due to physical disturbances (e.g., repeated clam digging and fishing, overload of aquaculture activities) and pollution by discharge effluent waters into the embayment (Chen et al., 2015). In our study, the two plots were set in contrasting habitats. Plot A was in the fragmented area affected by polluted effluents from aquaculture ponds and severe clam digging and fishing, consisted of isolated patches with a diminished seagrass cover (41.43 ± 7.02%). Plot B was in the continuous area with higher seagrass cover (85.21 ± 20.02%). These two plots, separated by <1 km, originally belonged to a big continuous seagrass meadow before serious disturbance. We hypothesize since they were once a part of a single meadow that there should be little genetic differences between these two plots. This may be dependent on the dispersal capacity of *E. acoroides*.

**Figure 2:**
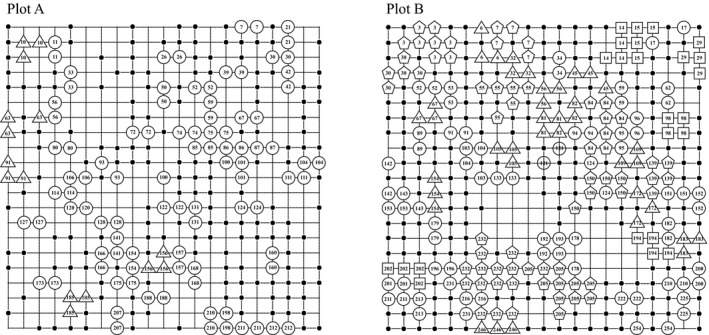
Genotype distribution of the two 20 × 20 m *Enhalus acoroides* plots in Li'an Lagoon. The sampling interval is 1 m. Points indicate single‐ramet genotypes; circle, triangle, and square represent the clone has 2, 3, and 4 ramets, respectively; pentagon indicates that the clone contains more than five clonal replicates. The absence of points or numbers indicates gaps in the plot. The numbers are codes of clones

Samples were regularly collected according to the grids with the interval of 1 m in each plot (Figure 2). In total, 271 and 395 shoots were collected in plot A and plot B, respectively. After thoroughly cleaning the mud and epiphytes, leaves were dried and preserved in silica gel.

### 2.2 Microsatellite amplification and genotyping

Total genomic DNA was extracted from 0.03 to 0.05 g dry leaves of *E. acoroides* using Plant Genomic DNA Kit (Tiangen, Inc., Beijing, China). Eight microsatellite loci were selected to genotype the samples (Gao, Jiang, Geng, & Chen, 2012; Nakajima et al., 2012), including Eaco_008, Eaco_009, Eaco_010, Eaco_051, Eaco_054, Eaco_055, EA447, and EA1461. Fluorescently labeled PCR reactions were carried out following Gao et al. (2012). Lengths of PCR products were analyzed by Gene‐Mapper 4.0 (Applied Biosystems).

### 2.3 Genetic statistics

Genetic variation of the clonal plants contains two parts: (a) genotypic diversity, the number, and evenness of genets and (b) traditional gene diversity, that is, allele number and heterozygosity. The number of multilocus genotypes (MLGs) was calculated using the R package “RClone” (Bailleul, Stoeckel, & Arnaud‐Haond, 2016) in R v3.4.1 (R Core Team, 2017). To determine the true clones, the probability, of identical MLGs deriving from distinct reproductive events (*P*_sex_) and its significance, were tested using the R package “RClone” with 1,000 permutations. Genotypic diversity within each plot was characterized by the number of distinct clones (*G*) and clonal richness (*R*) according to Dorken and Eckert (2001). Genetic diversity within each plot was indicated with the following parameters: number of alleles (*N*_a_), expected heterozygosity (*H*_E_), and inbreeding coefficients (*F*_IS_), which were calculated by FSTAT2.9.3.2 (Goudet, 1995). Genetic differentiation parameter *F*_ST_ between the two plots was estimated using FSTAT2.9.3.2.

### 2.4 Fine‐scale spatial genetic structure

Spatial genetic structure of *E. acoroides *was detected by spatial autocorrelation analyses performed at the ramet‐level (including all sampling units) and the genet‐level, respectively. For the genet‐level, spatial autocorrelation was conducted in two ways: (a) using central coordinates for each genet and (b) using one random geographic coordinates for each genet. The average kinship coefficients (*F_ij_*) for each distance class and the regression slopes (*b*) for kinship coefficient on geographical distance were estimated using GenClone v.2.0 (Arnaud‐Haond & Belkhir, 2007; Loiselle, Sork, Nason, & Graham, 1995). Ten distance classes were fixed in our study: 0–1, 1–2, 2–3, 3–4, 4–5, 5–8, 8–12, 12–16, 16–20, and 20–30 m. The significance of SGS pattern was tested by random permutation with 10,000 repetitions. The intensity of SGS in each plot was described by the statistic *Sp* following the formula:

$$Sp=-\frac{b\log}{1-F_{\left(1\right)}}$$

where *b*log is the slope of the regression for kinship coefficient on the logarithm of geographical distance, and *F*_(1)_ is the average kinship coefficient in the first distance class (Vekemans & Hardy, 2004). Spatial autocorrelation analyses were also performed using the relationship coefficient (*r_ij_*) described by Smouse and Peakall (1999) for each plot. Furthermore, to assess the effect of clonality on SGS, we compared the SGS patterns between the ramet‐level and the genet‐level within each plot using nonparametric heterogeneity tests by GenAlex 6.5 software (Peakall & Smouse, 2006,2012). Comparisons between continuous and patchy plots were also conducted to infer the potential effect of disturbance. Single‐class (*t* ^2^) and multiclass (*ω*) test criteria and associated *p*‐values were calculated with 9,999 bootstraps.

### 2.5 Estimation of gene dispersal patterns

Both clonal growth and seeds/pollen dispersal contribute to the spatial genetic structure of *E. acoroides*. To assess their relative importance, we used the model of parent–offspring dispersal variance (*σ* ^2^) proposed by Gliddon, Belhassen, and Gouyon (1987): *σ* ^2^ = $\sigma_{\text{sex}}^{2}/\sigma_{\text{veg}}^{2}$. The clonal growth dispersal variance ($\sigma_{\text{veg}}^{2}$) was calculated by the method described by Alberto et al. (2005):

$$\sigma_{\text{veg}}^{2}=\frac{1}{2}\times \frac{\sum_{i=1}^{N_{\text{G}}}\sum_{j=1}^{n_{j}}\frac{d_{\mathrm{ij}}^{2}}{n_{i}}+0.5^{2}\times \left(N-N_{\text{G}}\right)}{N}$$

*N* is the total number of genets; *N*_G_ is the number of genet with more than one ramets; *d_ij_* is distance between the ramet (*j*) and central coordinates of genet (*i*); *n_i_*is the number of ramets of genet (*i*); 0.5 is half of the minimum sampling intervals (1 m) in our study.

Total gene dispersal variance (*σ* ^2^) and neighborhood size (*Nb*) were estimated by SPAGeDi 1.3 (Hardy & Vekemans, 2002) following an iterative procedure using the central‐genet dataset. The effective population density (*D*_e_) was set to *D*⁄4, where *D* is the census population density (Hardy et al., 2006).

### 2.6 Flowering output

To assess the reproductive output and sex ratio of *E. acoroides*, we monthly verified all the shoots in the two plots for the occurrence of male and female flowers from June to December in 2017, respectively. Sex ratio is defined as male/female.

## 3 RESULTS

### 3.1 Genetic variation

In our study, 214 and 255 distinct microsatellite multilocus genotypes (MLGs) were identified as genets in plot A and plot B, respectively. Clonal richness values of plot A and plot B was 0.789 and 0.645, respectively. A total of 51 alleles were found in the two plots using eight microsatellite loci, 44 and 49 alleles for plots A and B, respectively. Allelic richness was similar across plots with 5.50 (plot A) and 5.93 (plot B). The values of expected heterozygosity were similar between the two plots at both the ramet‐level (plot A: 0.494; plot B: 0.490) and the genet‐level (plot A: 0.496; plot B: 0.492). Both plots showed significant heterozygote excesses at both the ramet‐level and genet‐level (Table 1). The genetic differentiation value (*F*_ST_ = 0.002) between these two plots was very small.

**Table 1:** Number of alleles (*N*_a_), expected heterozygosity (*H*_E_), and inbreeding coefficient (*F*_IS_) at ramet‐level and genet‐level of *Enhalus acoroides *at Li'an lagoon

Loci	Plot A					Plot B				
	*N*_a_	Ramet‐level		Genet‐level		*N*_a_	Ramet‐level		Genet‐level	
		*H*_E_	*F*_IS_	*H*_E_	*F*_IS_		*H*_E_	*F*_IS_	*H*_E_	*F*_IS_
Eaco_008	5	0.163	**−0.015**	0.187	**−0.022**	5	0.126	**0.019**	0.156	**0.024**
Eaco_009	4	0.603	**−0.106**	0.602	**−0.077**	4	0.603	**0.011**	0.609	**0.016**
Eaco_010	2	0.500	**−0.084**	0.499	**−0.094**	2	0.476	**−0.120**	0.484	**−0.067**
Eaco_051	9	0.660	**−0.044**	0.655	**−0.075**	9	0.637	**−0.100**	0.633	**−0.089**
Eaco_054	8	0.527	**−0.020**	0.540	**−0.044**	10	0.546	**−0.032**	0.532	**−0.023**
Eaco_055	8	0.598	**−0.041**	0.598	**−0.044**	10	0.602	**−0.033**	0.584	**0.008**
EA447	5	0.479	**0.000**	0.474	**0.027**	5	0.546	**−0.094**	0.545	**−0.048**
EA1461	3	0.421	**−0.033**	0.416	**−0.042**	4	0.382	**−0.020**	0.396	**0.012**
Multilocus	44	0.494	**−0.043**	0.496	**−0.051**	49	0.490	**−0.053**	0.492	**−0.026**

Note

Significant values are in bold (*p* < 0.05).

### 3.2 Clonal structure

**Figure 3:**
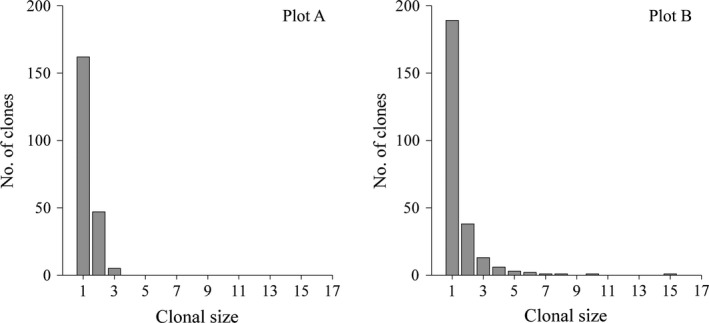
Frequency distribution of clonal size in the two *Enhalus acoroides* plots. Clonal size is represented by the number of clonal replicates belonging to the same clone

The clone distribution maps showed that most of the genets were represented by a single ramet in the two plots (Figure 2). The proportion of single‐genet was similar in plot A (75.7%) to that of plot B (74.1%), resulting in a highly skewed distribution of clone size (Figure 3). However, the overall distribution of clone size was very different between the two plots. In plot A, the largest clone had only three ramets. In contrast, 28 clones (10.9% of the genets) contained more than three ramets and the largest clone covered about 15 m^2^ in plot B.

### 3.3 Spatial autocorrelation

**Figure 4:**
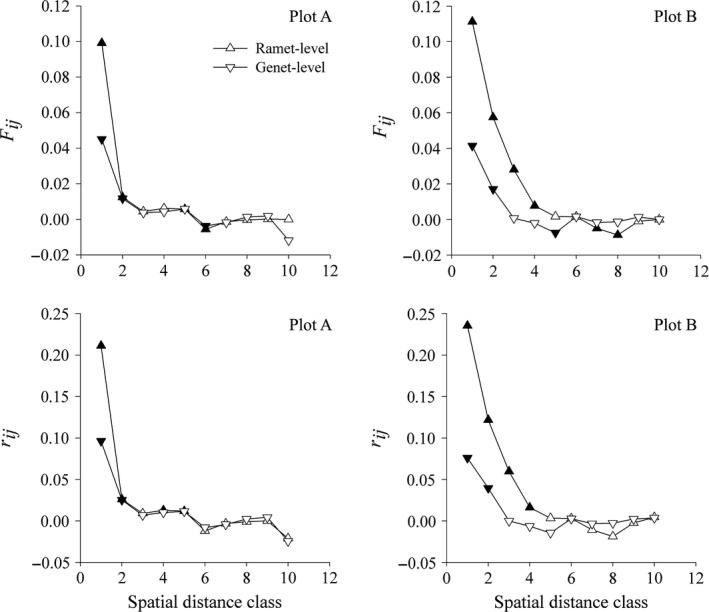
Fine‐scale spatial genetic correlograms of *Enhalus acoroides* for the two plots. The first row shows that the *y*‐axis of the correlogram is the mean pairwise kinship coefficient. In the second row, the *y*‐axis of the correlogram is the relationship coefficient. The significant values are presented by solid upper triangles and lower triangles for the ramet‐level and the genet‐level (central coordinates), respectively

Significant SGS patterns were observed both at the ramet and the genet‐levels in the two plots, and the intensity values *Sp* were stronger at the ramet‐level than the central‐genet‐level due to clonality (Table 2; Figure 4). In plot A, significant positive autocorrelation existed in 0–2 m at both the ramet and the genet‐levels. Significant heterogeneity was found between the ramet‐level and the genet‐level in the first distance class (Table 3, *p* = 0.01), but not in multiclass level (Table 3, *p* = 0.765). In plot B, significant positive autocorrelation occurred in 0–4 and 0–2 m at the ramet‐level and the genet‐level, respectively (Figure 4). Furthermore, correlograms of the ramet‐level and the genet‐level SGS were significantly heterogeneous in multiclass level (Table 3, *p* < 0.01). Comparing the two plots, significant heterogeneity was found at the ramet‐level, but not at genet‐level (Table 3).

**Table 2:** Results of the spatial autocorrelation for *Enhalus acoroides* based on kinship coefficient at different clonal levels

	*F*_(1)_	*b*log (±*SD*)	*Sp*	*Nb*
Plot A				
Ramet‐level	0.099***	−0.016 ± 0.001***	0.018	55.9
Central‐genet‐level	0.045***	−0.011 ± 0.002***	0.012	84.1
Random‐genet‐level	0.037	−0.010 ± 0.0001	0.010	101.6
Plot B				
Ramet‐level	0.111***	−0.024 ± 0.001***	0.027	36.8
Central‐genet‐level	0.041***	−0.008 ± 0.002***	0.008	126.8
Random‐genet‐level	0.037	−0.007 ± 0.0004	0.007	145.1

Notes

The mean kinship value of the first distance class *F*_(1)_. The regression slope (*b*log), *Sp* statistic, and the estimated neighborhood size (*Nb*).

*** *p* < 0.001.

**Table 3:** Comparison of spatial genetic structure for the two *Enhalus acoroides* plots using different data sets

Analysis levels	Distance class (intervals)										Total *ω*
	1	2	3	4	5	6	7	8	9	10	
	0–1 m	1–2 m	2–3 m	3–4 m	4–5 m	5–8 m	8–12 m	12–16 m	16–20 m	20–30 m	
Ramet versus Genet‐level											
Plot A	**6.927**	0.002	0.028	0.050	0.002	0.525	0.067	0.398	0.445	0.075	7.603
Plot B	**19.625**	**16.888**	**19.709**	**4.346**	**4.052**	0.008	3.270	**14.888**	0.840	0.008	**45.910**
Plot A versus Plot B											
Ramet‐level	0.595	**25.154**	**17.310**	0.103	1.088	**10.943**	**4.051**	**20.503**	0.226	**7.379**	**45.469**
Genet‐level	0.295	0.485	0.215	1.599	**5.647**	3.101	0.041	0.917	0.092	**5.340**	15.317

Note

Significant values are in bold (*p* < 0.05).

### 3.4 Indirect dispersal estimates

The estimated variance of clonal growth dispersal ($\sigma_{\text{veg}}^{2}$) was 0.138 and 0.170 in plot A and plot B, respectively. Within the sampled area, the variance of the gene dispersal (*σ* ^2^) converged successfully after iteration for plot A, but not for plot B. Estimated mean value of *σ* ^2^ was 10.960 in plot A. Consequently, the relative importance of the sexual and clonal growth for gene dispersal ($\sigma_{\text{sex}}^{2}/\sigma_{\text{veg}}^{2}$) was 79.420.

### 3.5 Flowering output

**Figure 5:**
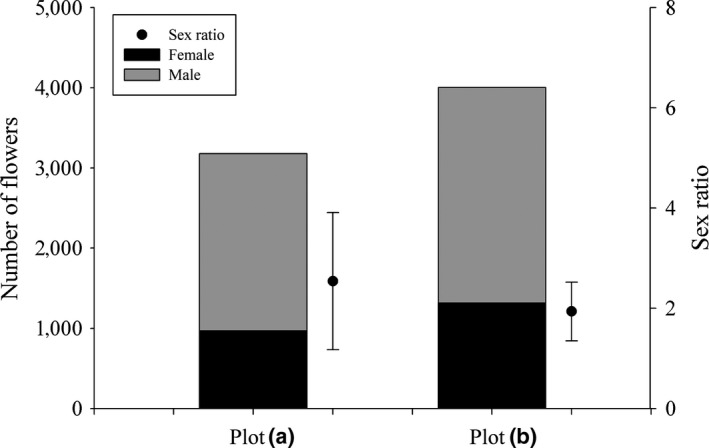
Comparison of flowering output and sex ratio between the patchy plot A and continuous plot of *Enhalus acoroides*

*Enhalus acoroides* had a male‐biased sex ratio at the ramet‐level during June to December in the two plots. The total number of flowers was higher in plot B than in plot A, and the sex ratio was more stable monthly in the continuous plot B (1.271–2.802) than in the patchy plot A (0.902–4.907; Figure 5).

## 4 DISCUSSION

### 4.1 Contribution of clonal growth and sexual reproduction

This study described an important role of sexual propagation in a nonobligate clonal marine plant. Sexual reproduction has long been considered to be less important than clonal growth for seagrass propagation, due to the occurrence of large clones (Reusch, Boström, Stam, & Olsen, 1999). However, there is a growing recognition that recruitment by seeds is also important in maintaining seagrass populations, as revealed by high clonal diversity (Ruggiero, Reusch, & Procaccini, 2005) or visible seed production and recruitment events in the field (Balestri, Vallerini, & Lardicci, 2017; Xu et al., 2018). In this study, high clonal diversity was found in both plots using a short sampling interval of 1 m, which was slightly lower than the previous population level studies (Yu et al., 2018). Olesen et al. (2004) also found that seeds of *E. acoroides* dominated the recolonization process in a disturbed seagrass meadow. These results highlight the potential importance of seedling recruitments for the persistence of *E. acoroides* populations.

The estimated sexual gene dispersal variance was about seventy‐nine times larger than clonal growth in the fragmented area, suggesting that gene dispersal mediated by sexual reproduction occurs along larger distances than that mediated by clonal growth. This result is in accordance with the reproductive strategy of *E. acoroides*. The rhizome elongation of *E. acoroides* is slow, ca. 3–10 cm/year (Marbà & Duarte, 1998; Thorhaug & Cruz, 1986), while the occurrence of sexual reproduction is relatively high (Duarte et al., 1997), and the floating ability of especially fruits but also seeds can be up to several kilometers, driven by water movements (Kendrick et al., 2012; Lacap et al., 2002). Genetic connectivity in this bay was evident indicated by the low genetic differentiation between the two locations. Such a phenomenon is probably due to the seed dispersal of *E. acoroides*. However, the contribution of clonal growth in this species appears to be underestimated in our study,because clonal size may be decreased by disturbances in plot A. The accurate dispersal patterns of pollen or seeds for *E. acoroides* will require further studies using parentage analysis.

### 4.2 Spatial genetic structure

The SGS strength estimated by the *Sp* statistic was higher at the ramet‐level than the genet‐level, indicating that clonality increases the SGS patterns of *E. acoroides*. Such patterns were also found in other seagrass species, for example *Cymodocea nodosa* (Alberto et al., 2005; Ruggiero et al., 2005) and *Zostera marina* (Hämmerli & Reusch, 2003a). The effect of clonality on fine‐scale genetic structure is reflected in the size of the clonal subrange (the largest geographic distance between two ramets belonging to the same clone). Beyond this spatial scale, clonality does not affect genetic structure. The clonal subrange of *E. acoroides* is shorter (4.2–10.2 m) than other seagrass species, such as *C. nodosa* (20–35 m, Alberto et al., 2005), *Posidonia oceanica* (12.7–76.6 m; Diaz‐Almela et al., 2007), and *H. ovalis *(8.9–40.1 m; N. N. Xu et al., unpublished data). Therefore, the pronounced effect of clonality on SGS only exists over several meters for *E. acoroides *in this study.

Limited dispersal of seeds/pollen is the key factor causing the significant fine‐scale SGS of seagrasses at the genet‐level (Hämmerli & Reusch, 2003a; Van Tussenbroek, Montero, Wong, Santos, & Guzman, 2010; Van Tussenbroek et al., 2016). For example, most of the *Z. marina* seeds disperse <5 m (Orth, Luckenbach, & Moore, 1994). The average dispersal distance of pollen and seeds for *Thalassia testudinum* were also short with about 0.3–1.6 and 0.3–0.4 m, respectively (Van Tussenbroek et al., 2016), although the fruits can float for kilometers. In our study, significant SGS occurred in 0–2 m for *E. acoroides* at the genet‐level. However, based on ecological evidence, it is not expected that the observed fine‐scale SGS of *E. acoroides* would arise from limited dispersal of seeds. In the field, we found that most of the naturally dehisced seeds float away from the mother plants immediately until the membranous seed coat has withered, and only then, they sink into the water. The distances between settled seeds and their mother plants can be much larger than 2 m. An alternative explanation is that the settlement distances among siblings from a single fruit are short. The seeds in a fruit cohort disperse at the same time and site. While following the direction of prevailing currents, seeds in a cohort fall to the bottom within short distance intervals, resulting in an aggregated distribution of seedlings, which increases the SGS. The peculiar pollen dispersal of *E. acoroides* may also contribute to this SGS pattern observed in *E. acoroides*, through the similar effect of pollen cohort dispersal aggregated from male flower. Pollen limitation is considered common in seagrasses species as rapid dilution in seawater which causes pollination to occur between neighbors (Van Tussenbroek et al., 2016). *Enhalus acoroides* is the only exception to hydrophilous pollen dispersal in seagrasses (Ackerman, 2006). One male spathe of *E. acoroides* can release hundreds of floating male flowers, and pollination can occur on the water surface when they encounter female flowers (Cox, 1988; Ackerman, 2006; S. Yu, personal observation). This unique mechanism, despite promoting large‐scale gene flow by dispersing longer distances, is likely to also contribute to SGS due to aggregated dispersal of pollen cohorts.

### 4.3 Potential effects of disturbance on flowering output and SGS patterns

Anthropogenic disturbance is the primary driving factor for global seagrass loss and degradation. At the population level, serious disturbance has directly decreased the cover and increased the shoot mortality in Li'an lagoon. The cover of *E. acoroides* was already reduced by half at the location near plot A compared with the data in 2008 (Chen et al., 2015). The total number of flowering shoots is likely to be positively correlated with seagrass cover and clone size (Hämmerli & Reusch, 2003b; Vermaat et al., 2004; S. Yu et al. unpublished data). As a result, the sexual reproduction of *E. acoroides* theoretically could be dramatically influenced by decreased density and decreased vigor. Our results indicated that the total flowering output of the patchy plot was much lower than the continuous plot. A similar pattern also showed in *Z. marina *meadows (Hämmerli & Reusch, 2003a; Reusch, 2003). However, the fruit set rate in the patchy plot A was higher than the continuous plot B (S. Yu et al., unpublished data), indicating that number/density of flowering shoot is not the only factor determining the seed set. Vermaat et al. (2004) found that fruit set of *E. acoroides* increased dramatically with seagrass cover at about 50%, suggesting that the efficiency of pollen trapping is in nonlinear correlation with seagrass cover and density.

The change of genetic diversity is not always pronounced as a response to disturbance, because even low levels of gene flow can significantly alleviate the loss of genetic diversity. However, SGS appears to be more sensitive to disturbance (Aguilar et al., 2008; Wang et al., 2011). In this study, both plots showed high genetic and genotypic diversity due to the reproductive biology and repeated seedling recruitments of *E. acoroides*, as explained above. The weaker ramet‐level SGS pattern observed in the patchy plot may be partially explained by disturbance. Alberto et al. (2005) also found that the ramet‐level SGS for *C. nodosa* was weaker in the disturbed site than stable population. The patchy plot A is very close to the aquaculture ponds, and the effluents dumped from the ponds create harmful conditions (e.g., high epiphyte loads and sulfide poisoning) for the seagrass meadow resulting in a large number of dead shoots. In these conditions of lower density and biomass, the natural SGS pattern caused by ramet growth within a neighborhood becomes altered. The autocorrelation patterns at the genet‐level SGS were similar between the two plots, although the cover of the patchy plot was half the amount of the continuous plot. We supposed that seedling recruitments occurring in the opening gaps might have buffered the negative effects of disturbance on *E. acoroides* meadows, because considerable fruits and floating seeds were found through our phenology observation in the Li’ an population (S. Yu et al. unpublished data). However, more evidence is needed to confirm this hypothesis, because we have no genotypic diversity data within the two plots prior to disturbance.

## 5 CONCLUSIONS

In the present study, we found that *E. acoroides* exhibits high clonal diversity, indicating the importance of sexual reproduction for propagation. This also highlights that seed‐based restoration may be adequate in the disturbed meadows. Significant SGS pattern was found at the ramet‐level and the genet‐level. Stronger SGS pattern at the ramet‐level indicated that clonality increases the spatial genetic structure at a fine scale. Results of nonparametric heterogeneity test suggested that SGS at the ramet‐level was more sensitive to the disturbance that caused massive death of shoots near the existing aquaculture sites. The genet‐level SGS was likely to be shaped by numerous processes, such as pollen and seed dispersal mechanisms, seedling recruitment patterns, as well as the environmental factors. The ongoing anthropogenic impacts as an external force will negatively affect the SGS in the long term, although *E. acoroides* exhibits high reproductive output, which may help it to recover from or resist, moderate disturbance. However, seedlings have very slow rhizome elongation rates and may be particularly susceptible to pollution and physical disturbances, causing high mortality rate and consequently, decreasing their capacity for long‐term maintenance and for recovery from perturbations. In summary, our results will be useful for the future restoration of *E. acoroides*.

## CONFLICT OF INTEREST

None declared.

## AUTHOR CONTRIBUTIONS

SY: designed and analyzed the work; and wrote the manuscript; YC: collected the samples and extracted the DNA; EAS: wrote the manuscript with collaboration of SY; JP: participated in the design of the work and collected the samples in the field; ZJ and CH: extracted the DNA; LJ: helped to collect flowering data in the field; AT, edited and approved the final version; XP: participated in the design the work and approved the final version.

## Data Availability

Microsatellite data can be found at Dryad Digital Repository: https://doi.org/10.5061/dryad.sn1h87s.
